# Ancestral complexity and constrained diversification of the ant olfactory system

**DOI:** 10.1098/rspb.2025.0662

**Published:** 2025-04-30

**Authors:** Simon Marty, Antoine Couto, Erika H. Dawson, Neven Brard, Patrizia d'Ettorre, Stephen H. Montgomery, Jean-Christophe Sandoz

**Affiliations:** ^1^ IDEEV, Université Paris-Saclay, CNRS, IRD, Evolution Genomes Behaviour and Ecology, 91190 Gif-sur-Yvette, France; ^2^ Laboratory of Experimental and Comparative Ethology, Université Sorbonne Paris Nord, 93430 Villetaneuse, France; ^3^ School of Biological Sciences, University of Bristol, Bristol BS8 1TQ, UK

**Keywords:** evolution, social insects, nestmate recognition, cuticular hydrocarbon, Formicidae, olfaction

## Abstract

Communication is a cornerstone of social living, allowing the exchange of information to align goals and synchronize behaviour. Ants, a group of highly successful social insects, have heightened olfactory abilities that are integral to their evolutionary success. Essential for colony cohesion and cooperation, a female-specific olfactory subsystem processes information about nestmate recognition cues (cuticular hydrocarbons), including basiconic sensilla on the antenna and a cluster of specific glomeruli in the antennal lobe. While it has often been linked to ants’ social lifestyle, the evolutionary origins and phylogenetic distribution of this system remain unknown. We conducted a comparative exploration of the ant olfactory system across eight major subfamilies, integrating neuroanatomical, chemical and behavioural analyses. Our findings reveal that sophistication of the ant olfactory system has deep evolutionary roots. Moreover, antennal lobe investment is not associated with social traits such as colony size, polygyny or foraging strategies, but correlates with cuticular hydrocarbon profile complexity. Despite neuroanatomical differences, different ant species consistently excel in nestmate discrimination, indicating adaptation to chemical diversity while maintaining reliable social recognition. This suggests that cuticular hydrocarbon profile and neuronal investment in olfactory neuropil have co-evolved to sustain discrimination performance.

## 1. Introduction

Effective communication is fundamental to the success of social living. It enables group members to exchange information, resolve conflicts and coordinate their actions, allowing successful cooperation and group cohesion. Consequently, whether in mammalian societies or vast colonies of social insects, social evolution is believed to influence the development of recognition and communication systems, thereby supporting reliable social interactions [1–4]. Despite significant progress in understanding the neural components of communication systems, particularly in social insects [5], a notable knowledge gap remains regarding the evolution of the sensory systems that support this critical function. Therefore, exploring the roles of novel neuronal populations and other adaptations within the sensory pathways that support communication is key to understanding the mechanisms underlying the evolution of social behaviour.

Ants stand as prominent models of group living and cooperation, owing to their remarkably large and complex colony organizations, diverse kin structures and extensive interspecific variation in morphological traits, dietary preferences, foraging behaviours and life history strategies [6,7]. At the core of their cooperative behaviours lies a sophisticated communication system that supports pheromonal signalling and the perception of recognition cues, primarily odourant compounds detected by the olfactory system [8,9]. Accordingly, ants possess one of the most complex olfactory systems among insects [10–12], as evidenced by studies on selected species which highlighted its pivotal role in social interactions and scent-guided behaviours [13,14]. Nevertheless, a significant gap remains regarding the evolutionary trajectory of ants’ olfactory system. Understanding these trajectories could illuminate the adaptive significance of olfaction within the context of sociality and the remarkable taxonomic radiation of ants.

Insect antennae are typically covered with different types of sensory hairs known as sensilla, which enclose the dendrites of olfactory sensory neurons (OSNs). The axons of these neurons project to the antennal lobe (AL) in the brain, where they form glomeruli, discrete spheroidal structures that serve as processing units. Each OSN generally expresses a single olfactory receptor (OR)—together with the ubiquitous co-receptor—which defines its response profile to odourant stimuli [15,16]. The OR expression also dictates the specific glomerular target of each neuron, establishing a nearly one-to-one correspondence between OR numbers and glomerular count [17]. These olfactory glomeruli serve as central hubs where local interneurons and neuromodulatory neurons refine olfactory information before it is relayed to higher brain centres by projection neurons [18].

The complexity of ant olfactory systems is supported by two key observations. First, ants possess a high number of AL glomeruli and a greater abundance of associated OR genes compared with most other insect clades [10,11,15,19]. Second, ants exhibit an olfactory specialization that has been associated with the detection of recognition cues [5]. This specialized pathway is characterized by a unique type of antennal sensilla, the basiconic sensilla, wherein OSNs express a specific clade of 9-exon ORs [11] and project exclusively to a distinct cluster of glomeruli [20,21], which lack serotonergic innervation [22–25]. Notably, electrophysiological recordings have revealed that cuticular hydrocarbons (CHCs), which are identity-signalling compounds, are sensed by the 9-exon ORs [26–28] and basiconic sensilla OSNs [29–33]. These molecules are pivotal in insect communication [34], conveying information about species, colony affiliation, life stages and reproductive status [35], thereby facilitating the recognition of nestmates over non-nestmates and the maintenance of colony cohesion [36]. Thus, this olfactory subsystem is believed to have evolved and expanded significantly in ants, driven by the need to meet sophisticated communication demands within complex social colonies [11,19,28,37].

At this stage, our knowledge of ants’ olfactory system is biased towards a few specific clades, significantly limiting insight into how its sophistication relates to ants’ social behaviour and ecology. For example, it is not yet known whether the increased complexity of the AL and the extensive repertoire of ORs translate into enhanced accuracy in recognizing social identities, such as distinguishing between nestmates and non-nestmates. Additionally, the diversification of recognition cues, particularly the chemical composition and complexity of CHC profiles [38,39], likely interplays with the evolution of the ant olfactory subsystem. This co-evolution suggests a dynamic feedback mechanism in which increasingly complex chemical signals drive adaptations within the neural and sensory apparatus. However, to date, little is known about the influence of diverse communication demands and varied social structures on the evolutionary trajectory of the olfactory system across the ant phylogeny [40]. Therefore, it remains uncertain whether the olfactory system was already complex at the onset of ants’ remarkable taxonomic radiation or if its complexity evolved during their diversification.

Here, we employed a comparative approach to explore the evolutionary history of key olfactory neuroanatomical traits in ants, examining whether variation in ecology and social structure have influenced differential investment in their olfactory system. Overall, we investigated 14 species from the eight most species-rich Formicidae subfamilies [41], differing in feeding behaviour, ecological niche and colony kin structure. We compared the distribution of basiconic sensilla across antennal segments and examined the AL glomeruli that receive serotonergic innervation, aiming to identify those involved in social recognition. Additionally, we analysed neuropil volumes and glomerular counts, integrating these measurements to provide a comprehensive assessment of the olfactory system in ants. We conducted ancestral state reconstructions and evolutionary rate analyses on the number of glomeruli to identify derived traits and periods of rapid evolution. Using this evolutionary framework, we finally tested whether the anatomical variation observed in the olfactory system correlates with behavioural differences between three exemplary species, and is linked to ecological or chemical factors. By integrating these diverse approaches, we aimed to elucidate the role of ecological and/or social factors as selection pressures shaping the evolution of the olfactory system across the ant phylogeny.

## 2. Results

### (a) Diversity and distribution of antennal sensilla

**Figure 1.**
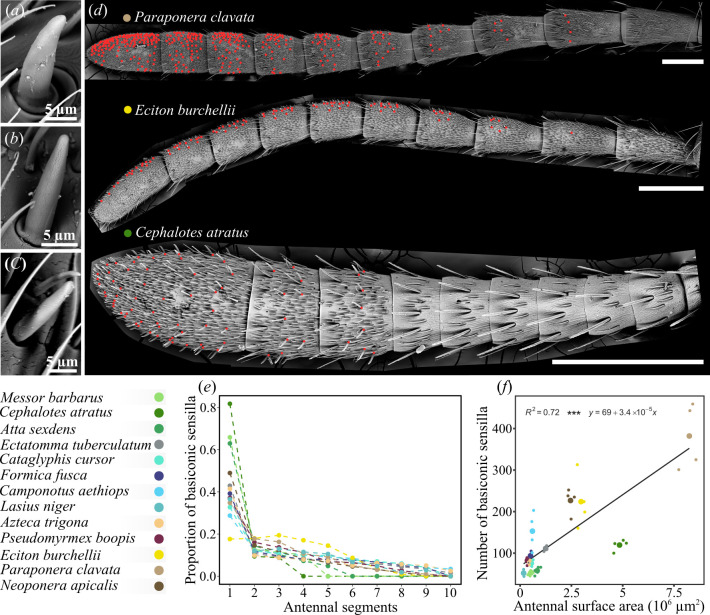
Distribution of basiconic sensilla on ant antennae. (*a–c*) Scanning electron micrographs of basiconic sensilla across ant species. These sensilla show slight morphological differences, including a reduction in base thickness from basal taxa (*Neoponera apicalis*; *a*) to Formicinae (*Formica fusca*; *b*) and Myrmicinae clades (*Cataglyphis cursor*; *c*). (*d*) Scanning electron micrographs of *Paraponera clavata*, *Eciton burchellii* and *Cephalotes atratus* antennae, illustrating the distribution of basiconic sensilla (red dots) across antennal segments. These sensilla are more densely packed on the distal segments of the antenna. Scale bars, 0.5 mm. (*e*) Relative proportion of basiconic sensilla across antennal segments in various ant species (coloured dots). Basiconic sensilla are notably concentrated in the distal part of the antennae, prominently in Myrmicinae (varying green shades). (*f*) Number of basiconic sensilla on the antenna plotted against antenna surface area, showing species means (large coloured dots) and individual data points (small dots). The number of basiconic sensilla correlates strongly with the size of the antenna (Pearson test: *t* = 4.97, d.f. = 11, ***p < 0.001, *R*² = 0.72).

To explore the evolution of sensory structures in ants, we conducted scanning electron microscopy on the antennae of 13 ant species spanning a broad phylogenetic range. Among the various morphological types of sensilla, basiconic sensilla, identified by their peg-in-socket shape and porous tip, contrast with other sensory hairs [42]. This sensillum type was consistently present across all studied species, exhibiting only slight morphological differences (figure 1*a*–c).

In ants, the distribution of basiconic sensilla is strongly biased towards the distalmost segments of the antennae (figure 1*d,*.e; two-way ANOVA, *p* < 0.001 across flagellomeres), with their proportion decreasing towards proximal segments. Consequently, there is a progressive decline in both the number and density of sensilla along the antenna (electronic supplementary material, figure S1d,e), albeit with significant variability in attenuation patterns across species (figure 1*e*; two-way ANOVA, *species* × *segment* interaction, *p* < 0.001; electronic supplementary material, figure S1d,e). The decline is particularly pronounced in Myrmicinae species, where basiconic sensilla are absent beyond the third segment in *Cephalotes atratus*, the fourth segment in *Atta sexdens*, and the fifth segment in *Messor barbarus*. By contrast*, Eciton burchellii* (Dorylinae) exhibits a more even distribution of basiconic sensilla across the first four segments, with each containing approximately 18% of the total count (figure 1*d*,*e*; *post hoc* Tukey, *p* > 0.05 between segments 1, 2, 3 and 4). Across species, the total number of basiconic sensilla varies significantly (electronic supplementary material, table S1 and figure S1e; two-way ANOVA, *p* < 0.001), ranging from 52.2 ± 5.6 in *M. barbarus* to 382 ± 80 in *Paraponera clavata*. These differences primarily reflect variation in antenna size, as indicated by the strong correlation between basiconic sensilla counts and the measured antennal surface area (figure 1*f*; Pearson test, *t* = 4.9664, d.f. = 11, *p* < 0.001, *R*² = 0.72).

### (b) Characterization of anatomical regionalization in the antennal lobe

**Figure 2.**
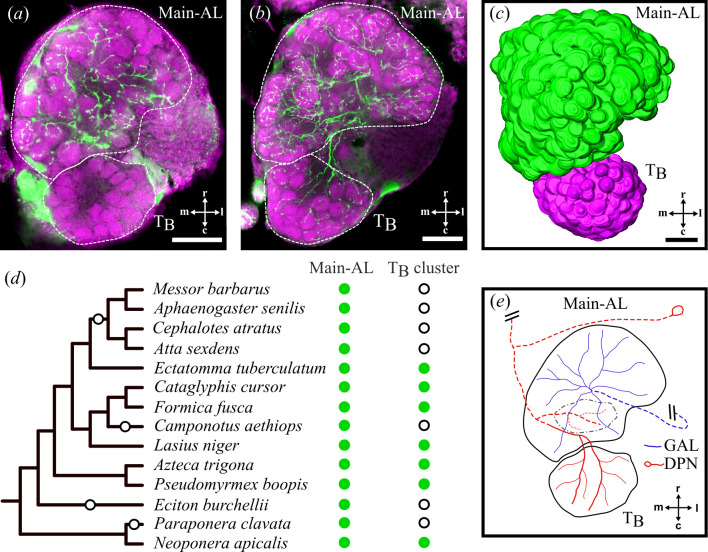
Anatomical characterization of the antennal lobe (AL). (*a,b*) Confocal optical sections of the antennal lobe in an ant species lacking serotonin innervation in the T_B_ cluster (*a*; *Messor barbarus*) and a species with serotonin immunoreactivity in this region (*b*; *Ectatomma tuberculatum*). The glomeruli are stained with hydrazide-conjugated dye displayed in magenta, and immunolabelled serotonergic projections are displayed in green. The AL subregions are outlined with dashed lines. (*c*) 3D reconstruction of glomerular volumes and antennal lobe regionalization in *Neoponera apicalis*. The main-AL, which receives serotonergic innervation, is shown in green, while the T_B_ cluster, which lacks such innervation in some species, is in magenta. (*d*) Variation in serotonergic innervation across ant species summarized on the phylogeny. Green dots indicate serotonergic innervation, while empty circles in the T_B_ cluster column represent the absence of such projections. Losses of serotonergic projections, predicted by the most parsimonious scenario, are marked with empty circles on the tree branches. (*e*) Diagram summarizing serotonergic projections in the AL of ants with serotonergic T_B_ clusters. In all species, the giant neuron innervating the AL (GAL, in blue) innervates the main-AL, while the deutocerebral projection neuron (DPN, in red) targets the dorsal cluster of glomeruli (black dashed line). In species with immunoreactive T_B_ clusters, DPN extends an additional branch to innervate the T_B_ glomeruli. Dashed lines represent structures that are dorsal relative to the AL. All scale bars represent 50 μm (r, rostral; c, caudal; m, medial; l, lateral).

Given the marked variation in sensilla numbers, despite a consistent organizational pattern, we investigated how these differences affect the structure of the antennal lobe (AL). Using immunohistochemistry and confocal microscopy, we characterized the AL across 14 species of Formicidae. In all examined species, the AL exhibits two distinct regions resembling segregated glomerular clusters (figure 2*a*–c). The dorso-caudal region typically features more compact glomeruli compared with the rostral region of the AL (referred to as the main-AL, figure 2*a*–c). This organization was consistently observed across species, along with additional shared characteristics that define the T_B_ cluster [20,21]. A detailed description of the criteria used to delineate the T_B_ cluster is provided in the supplementary results (electronic supplementary material, figure S5 and §S3.2, Definition of the TB cluster), along with movies of AL scans and three-dimensional (3D) reconstructions [43]. This cluster, previously termed T6 in ants owing to the numbering of its input tract [25,44], was renamed T_B_ to ensure consistency across Hymenoptera [5,45], as its relative position varies among taxa (e.g. T3 in bees [46], T8 in hornets [45,47]). In ants and hornets, basiconic sensilla OSNs project exclusively into this cluster [20,21,47], which is reported to lack serotonin innervation in the *Camponotus* clade, unlike the rest of the AL, which is typically innervated by serotonergic neurons [22–24]. Lack of serotonergic innervation has been proposed as a marker for the T_B_ cluster [47]. Therefore, we investigated the innervation patterns of serotonergic neurons across the Formicidae, as a marker of functional and anatomical regionalization within the AL of ants.

We found that the T_B_ cluster lacks serotonergic neurons in *A. sexdens* (electronic supplementary material, figure S2a), *M. barbarus* (figure 2*a*), *C. atratus*, *Aphaenogaster senilis* and *Camponotus aethiops* (electronic supplementary material, figure S2b), while the rest of their ALs exhibit clear innervation (summarized in figure 2*d*). Similarly, the T_B_ cluster in *P. clavata* and *E. burchellii* appears to lack serotonergic fibres, although this observation is less certain owing to lower quality of our immunostaining replicates. By contrast, *Ectatomma tuberculatum* (figure 2*b*), *Neoponera apicalis* (electronic supplementary material, figure S2c), *Pseudomyrmex boopis* (electronic supplementary material, figure S2d), *Formica fusca*, *Lasius niger*, *Cataglyphis cursor* and *Azteca trigona* all exhibit clear serotonergic innervation in the T_B_ cluster (as summarized in figure 2*d*), with varying densities across both the main-AL and the T_B_ glomeruli.

We further traced and examined the innervation pattern of serotonin-immunoreactive neurites within the two subregions of the AL (figure 2*e*). In all species, the glomeruli of the main-AL primarily receive projections from a neuron known as the giant neuron (GAL), which originates in the suboesophageal zone and innervates the AL [23]. Additionally, the soma of another neuron, known as the deutocerebral projection neuron (DPN), is consistently observed within the lateral cell cluster near the rostral edge of the AL [23]. In all species, this DPN innervates a few glomeruli in the dorsal region of the main-AL and sends ipsilateral projections towards the mushroom bodies (figure 2*e*). However, in T_B_ immunoreactive species, we observed that the DPN exhibits an additional branch that specifically innervates the T_B_ cluster. Therefore, the absence of serotonergic innervation in T_B_ clusters correlates with the loss of this DPN extension, which can serve, along with morphological characteristics, as a key anatomical feature for characterizing T_B_ across species.

### (c) Volumetric relationships in the ant antennal lobes

**Figure 3.**
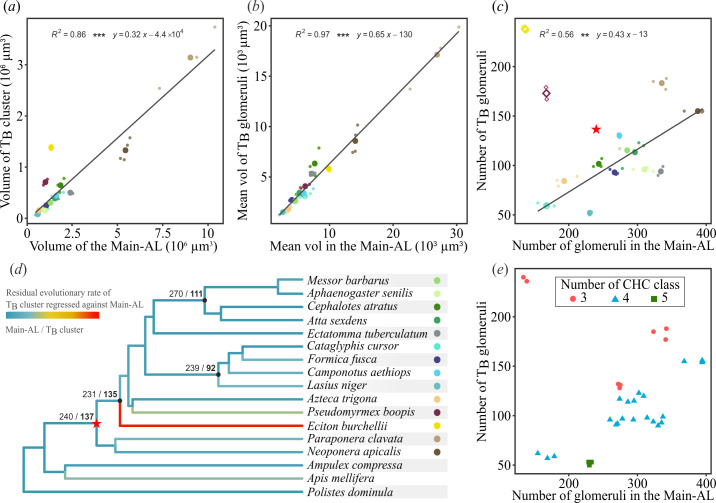
Evolutionary dynamics in the ant antennal lobe. (*a*) Volume of the T_B_ cluster plotted against the volume of the main-AL across different ant species. There is a significant correlation between the volumes of the T_B_ cluster and of the main-AL (*t* = 15.8, ***p < 0.001). (*b*) Mean volume of glomeruli in the T_B_ cluster plotted against the mean volume of glomeruli in the main-AL across different species. There is a correlation between the mean glomerular volumes in the two subregions of the AL (*t* = 33.9, ***p < 0.001), with T_B_ glomeruli being consistently smaller than those of the main-AL. (*c*) Number of glomeruli in the T_B_ cluster plotted against the number of glomeruli in the main-AL. These counts are not correlated (*t* = 0.70, *p* = 0.48). However, when excluding outlier species (squares), the counts significantly correlate (*t* = 6.61, **p < 0.01). The red star on the graph represents the putative position of ant’s most recent common ancestor (see text and electronic supplementary material, figure S3a–c). (*d*) Residual evolutionary rates of the number of glomeruli in the T_B_ cluster, relative to the main-AL, mapped onto the branches of the phylogenetic tree of the sampled species. The branches leading to *Eciton burchellii* and, to a lesser extent, *Pseudomyrmex boopis*, display high evolutionary rates, suggesting two independent expansions of the T_B_ cluster. Numbers indicated at the nodes represent the inferred ancestral state estimates for the number of glomeruli in both the main-AL and the T_B_ cluster. (*e*) Plot showing the number of glomeruli, similar to (*c*), with species categorized according to the number of compound classes (alkanes, alkenes, mono-, di- or tri-methyl alkanes) within their CHC profiles. There is a significant effect of the number of CHC classes on the proportion of glomeruli between the T_B_ cluster and the main-AL (*p*_MCMC_ < 0.01).

Using 3D models reconstructed from confocal image stacks (figure 2*c*), we measured the volume of the AL as the total glomerular area and found strong differences across species (electronic supplementary material, table S1 and figure S2e; Kruskal–Wallis test, *χ*^2^ = 38.9, d.f. = 14, *p* < 0.001). This is illustrated by the 20-fold difference in AL size between *L. niger* (0.69 × 10^6^ µm^3^) and *P. clavata* (12.1 × 10^6^ µm^3^), likely reflecting their substantial difference in body size. We also investigated neural investment in the two AL subdivisions, which are believed to serve different functions, by examining the scaling relationship between the volume of the main-AL and that of the T_B_ cluster. Despite considerable total volumetric variation, we found a significant correlation between the volumes of the main-AL and that of the T_B_ cluster (figure 3*a*; Pearson test, *t* = 15.8, *p* < 0.001, *R*^2^ = 0.86). This indicates that neuronal investment in the T_B_ cluster follows a relatively stable allometric relationship across ant species (electronic supplementary material, table S1).

We examined the relationship between the mean glomerular size within both subregions, by dividing each subregion’s volume by its number of glomeruli. Our analysis revealed a significant correlation between the volumes of glomeruli in the T_B_ cluster and those in the main-AL (figure 3*b**;* Pearson test, *t* = 33.9, *p* < 0.001, *R*^2^ = 0.97). The slope (0.65 [0.607, 0.684] with 95% CI) is significantly lower than 1 (*t-*test, *t* = −32.3, *p* < 0.001), with a negative *y*-intercept. This indicates that, across species, glomeruli of the T_B_ cluster are consistently smaller than those of the main-AL.

### (d) Glomerular counts and evolutionary dynamics in the ant antennal lobes

The glomerular count offers a measure of the AL’s computational power, with more glomeruli suggesting a greater capacity to integrate and discriminate diverse olfactory information through combinatorial processing [48]. Across our sample set, the number of glomeruli in the AL of worker ants varied significantly across species (electronic supplementary material, table S1 and figure S3a; Kruskal–Wallis test, *χ*^2^ = 38.5, d.f. = 14, *p* < 0.001), ranging from 227 ± 10 (mean ± s.d.) glomeruli in *C. cursor* to 543 ± 13 glomeruli in *N. apicalis*. These differences are evident within each AL subdivision, with significant variation in the number of glomeruli in both the T_B_ cluster (electronic supplementary material, table S1 and figure S3b,c; Kruskal–Wallis test, *χ*^2^ = 38.9, d.f. = 14, *p* < 0.001) and the main-AL (electronic supplementary material, table S1 and figure S3b,c; Kruskal–Wallis test, *χ*^2^ = 39.4, d.f. = 14, *p* < 0.001).

We further examined whether ants invest differently in the two AL subdivisions by studying the scaling relationship between the number of glomeruli in the T_B_ cluster and the main-AL. These counts are not correlated across all ants (Pearson test, *t* = 0.70, *p* = 0.48), owing to notable deviations of a few species (figure 3*c*). We therefore analysed the evolutionary rate of glomerular numbers using a variable-rate Brownian motion model across the ant phylogeny [49]. The tree was rooted using additional glomerular counts from the paper wasp *Polistes dominula* (electronic supplementary material, figure S2f), the emerald cockroach wasp, *Ampulex compressa* (electronic supplementary material, figure S2g), and the honeybee *Apis mellifera* ([50], see electronic supplementary material, Methods). We generated separate models for the two subregions, and displayed the T_B_ cluster residuals regressed against the main-AL along the tree to identify deviations in the allometric relationship between these subregions (figure 3*d*). Our analysis revealed a higher evolutionary rate on the branches leading to *E. burchellii* and, to a lesser extent, *P. boopis*, compared with the rest of the tree. These two species are notable outliers, with their T_B_ clusters comprising 63.3 and 50.8% of the total AL glomeruli, respectively. Excluding these species, the glomeruli numbers between the two AL subdivisions show a significant correlation (figure 3*c*; Pearson test, *t* = 6.61, *p* < 0.01, *R*^2^ = 0.56). This suggests that the variation in glomerular numbers is allometrically consistent between AL compartments, except in *E. burchellii* and *P. boopis*, which exhibit a disproportionately high number of T_B_ glomeruli. Incorporating literature data on *Ooceraea biroi* [11] and *Dolichoderus* sp. [51], we corroborate this trend and provide support for an expanded T_B_ cluster in the Dorylinae subfamily (electronic supplementary material, figure S3d,e).

We finally used maximum likelihood ancestral state estimation to reconstruct the evolutionary history of the AL across ants. The global model predicted approximately 367 AL glomeruli in the most recent common ancestor (MRCA) of ants (electronic supplementary material, figure S3a). Specifically, the analysis predicted 137 glomeruli for the T_B_ cluster (figure 3*d* and electronic supplementary material, figure S3b) and 242 for the main-AL (figure 3*d* and electronic supplementary material, figure S3c) in independent models. Contrary to previous conclusions [11,37], our findings suggest that the T_B_ subsystem was already elaborated before the extensive taxonomic radiation of ants. Furthermore, the model estimated 142 T_B_ glomeruli in the MRCA of Dorylinae and other formicoids, and 133 T_B_ glomeruli in the MRCA of *P. boopis* and the Dolichoderinae, indicating distinct expansions of T_B_ glomeruli relative to the main-AL in these lineages (figure 3*d* and electronic supplementary material, figure S3b,d). These expansions were accompanied by a reduction in the number of other glomeruli (electronic supplementary material, figure S3c), distinguishing these species from the general trend.

### (e) Social and chemical predictors of glomerular variation

We next aimed to elucidate the social and ecological correlates of the number of glomeruli in the T_B_ cluster and the main-AL using phylogenetically controlled mixed models, which incorporate social and chemical predictors (electronic supplementary material, table S2). First, we analysed social predictors, including types of polygyny (strictly monogynous versus facultatively or obligatorily polygynous), foraging strategies (solitary versus collective) and colony sizes (categorized as fewer than 1000, between 1000 and 10 000, and more than 10 000). None of these factors had any significant effect on the number of glomeruli (total, T_B_ or main-AL), the number of basiconic sensilla, or volumetric measures of the AL (total, T_B_ or main-AL) (see electronic supplementary material, Data file, tab mcmcGLMM). Likewise, these social factors did not affect the scaling of glomeruli between the T_B_ cluster and the main-AL (electronic supplementary material, figure S4; polygyny: *p*_MCMC_ = 0.388; foraging: *p*_MCMC_ = 0.827; colony size: *p*_MCMC_ = 0.386 and 0.387).

Next, we investigated whether variation in species-specific cuticular chemical profiles is related to these anatomical traits, using chemical analyses by gas chromatography coupled with mass spectrometry (see electronic supplementary material, §S1.7 Chemical analysis of CHC profiles). From each species’ chemical profile, we extracted three key variables: the number of individual CHCs (CHC number), the number of distinct CHC classes (ranging from 1 to 5, including alkanes, alkenes, mono-, di- or tri-methyl alkanes) and a measure of profile complexity, the Shannon index (SI; see §4).

All models that included the SI showed it as a significant predictor of the number of glomeruli in the T_B_ cluster (full model: posterior mean = −2.014, *p*_MCMC_ = 0.005). Specifically, CHC profile complexity was negatively associated with the number of T_B_ glomeruli, suggesting that species with less complex CHC profiles have more glomeruli in the T_B_ cluster. However, the inclusion of the SI only marginally improved the model fit compared with the model in which it was excluded (ΔDIC = 0.25, where DIC is the deviance information criterion).

Since the SI accounts for both the number of CHC compounds and the number of classes, we explicitly tested these parameters in the model. While the number of compounds did not significantly influence the number of T_B_ glomeruli (posterior mean = 0.0049, *p*_MCMC_ = 0.595), the number of CHC classes was negatively associated with this count (posterior mean = −0.827, *p*_MCMC_ = 0.045). This indicates that a lower diversity of CHC classes is associated with a higher number of T_B_ glomeruli, although the improvement in DIC was minimal when comparing the full model with the one without CHC class (ΔDIC = 0.05). However, none of the chemical variables (SI, CHC number, CHC classes) was a significant predictor of glomeruli numbers in the main-AL or of the total glomerular count (for SI, main-AL: posterior mean = −0.373, *p*_MCMC_ = 0.591; total: posterior mean = −0.873, *p*_MCMC_ = 0.0976). These chemical factors also did not significantly predict the volume of the AL (total, T_B_, or main-AL) or the number and density of basiconic sensilla. Investigating the scaling of glomeruli numbers between the T_B_ cluster and the main-AL, we again found SI to be significantly associated with investment in both olfactory regions (posterior mean = −1.924, *p*_MCMC_ = 0.0100).

In conclusion, models including chemical predictors were the most parsimonious and best-fitting, though they showed only a slight DIC improvement (ΔDIC < 1). Specifically, the chemical variables, SI and the number of CHC classes emerged as significant predictors of ants’ investment in glomeruli numbers in the T_B_ cluster.

### (f) Neuroanatomy’s impact on ants’ discrimination performance

**Figure 4.**
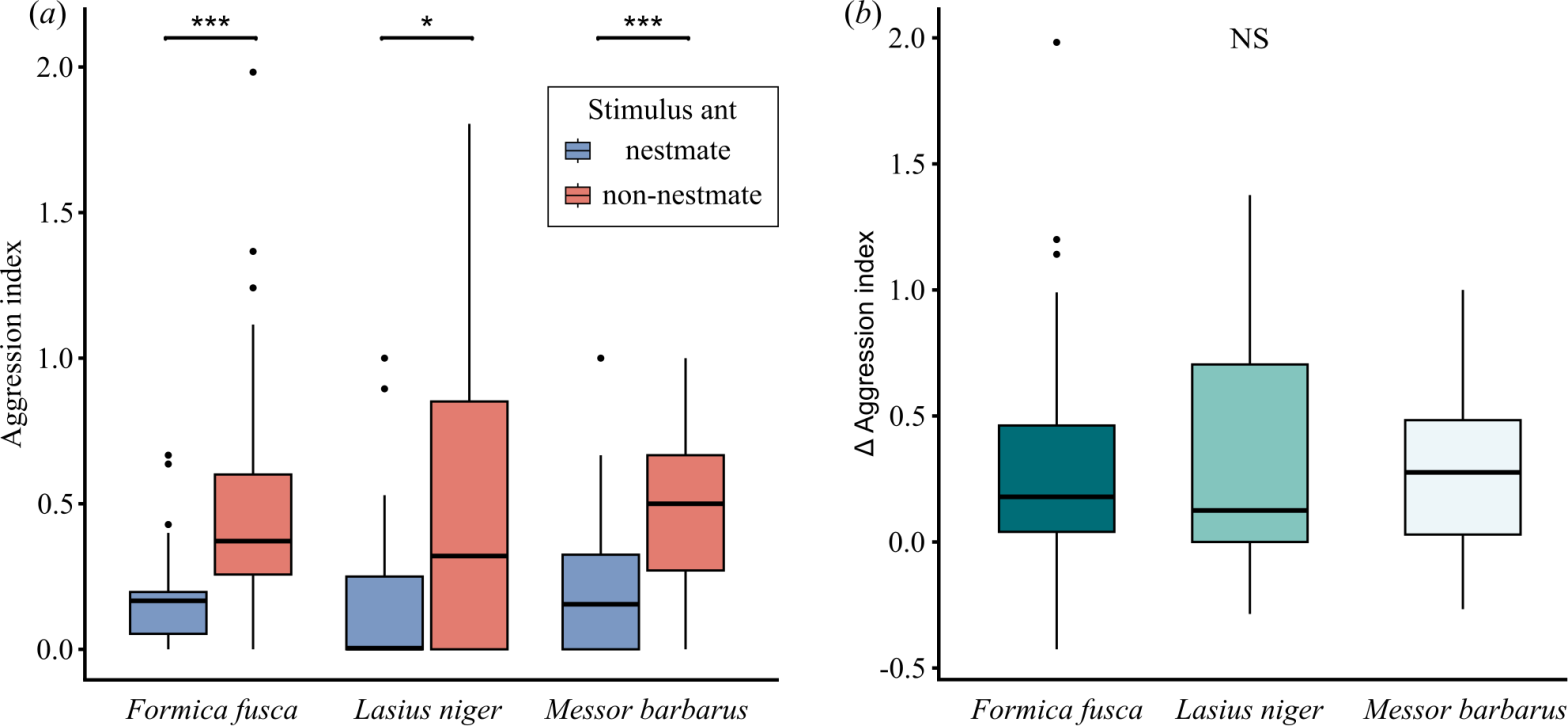
Comparative analysis of nestmate discrimination abilities. (*a*) Boxplots show median, quartiles and minimum/maximum values (whiskers) for aggression index. All investigated species discriminate nestmate from non-nestmate odours (***p < 0.001, *p < 0.01). (*b*) Boxplots represent median, quartiles and minimum/maximum values (whiskers) for delta aggression scores. Delta scores were calculated by subtracting the aggression index towards nestmates from that towards non-nestmates. Positive scores indicate more aggression towards non-nestmates, while negative scores indicate more aggression towards nestmates. The species were equally efficient at discriminating between nestmate and non-nestmate stimuli (NS: *p* > 0.05). In each boxplot, black circles indicate outliers. *n* = 30 individual ants for each species.

Building on the interspecific differences we found in serotonergic innervation and glomerular number, we tested whether this variation reflects differences at the behavioural level. We conducted a nestmate discrimination assay on a selected set of species: *M. barbarus* and *F. fusca*, which share approximately the same number of glomeruli in both subregions, and *L. niger*, which has a lower number of glomeruli in the T_B_ cluster compared with the other two species. Additionally, the T_B_ clusters of *F. fusca* and *L. niger* receive serotonergic innervation, while it is absent in *M. barbarus*. Workers of all these three species were able to perform a discrimination task, consistently showing higher aggression levels towards non-nestmates than towards nestmates (figure 4*a*; *F. fusca*: *χ*^2^ = 18.2, d.f. = 1, *p* < 0.001; *L. niger*: *χ*^2^ = 5.52, d.f. = 1, *p* = 0.0188; *M. barbarus*: *χ*^2^ = 13.0, d.f. = 1, *p* < 0.001). Overall, we found that the three species were similarly efficient at discriminating between nestmate and non-nestmate stimuli, with differentiation scores that did not significantly differ among species (figure 4*b*; *χ*² = 0.098, d.f. = 2, *p* = 0.95).

## 3. Discussion

In this study, we investigated anatomical variation within the olfactory system in a subset of ant species, encompassing all major taxonomic lineages of the Formicidae, to explore the evolution and adaptation of their CHC-related subsystem. We observed significant variation in olfactory traits, including the number of basiconic sensilla, as well as the volume and number of glomeruli in the antennal lobes (ALs). Despite this variation, the olfactory systems of these ant species exhibit a common ground plan characterized by a higher proportion of basiconic sensilla on the distalmost segments of the antennae, and consistent proportions of neuropil volume and glomeruli number between AL subregions. However, we also identified remarkable outliers, demonstrating a high evolutionary rate of T_B_ glomeruli within specific subfamilies, resulting in the expansion of the T_B_ cluster relative to the main-AL. Nonetheless, contrary to previous assumptions [11,19,37], ancestral traits reconstruction suggests that the olfactory system was already sophisticated in the most recent common ancestor (MRCA) of ants, with possibly 367 glomeruli, including 142 within the CHC-related subsystem. In considering ecological and chemical factors that might influence this evolution, the complexity of ants' CHC profiles, particularly CHC class diversity, emerged as a potential predictor of variation in glomerular investment, albeit with modest explanatory power. Nonetheless, neither the relative nor absolute glomerular investment appears to influence nestmate discrimination performance across species. Thus, our results suggest that ants possessed a sophisticated olfactory system prior to their taxonomic radiation, and subsequent evolution was likely shaped by the necessity to maintain high olfactory performance across diverse ecological conditions and life history strategies.

### (a) An olfactory subsystem for hydrocarbon sensing in ants

Ants universally bear a wide diversity of CHCs on their body surface, which serve as efficient recognition cues [52]. These compounds play a pivotal role in social communication, especially in nestmate recognition, which is crucial for preventing exploitation by competitors and parasites, and maintaining colony cohesion. Given that this task is typically carried out by female workers, the absence of basiconic sensilla and T_B_ glomeruli in males hints at their specialized function in detecting recognition cues [25,53–55]. This idea is further supported by electrophysiological studies demonstrating the role of basiconic sensilla OSNs [29,31,33], as well as expression-biased ORs of the 9-exon clade [11,56], in responding to CHCs [26,27]. While this subsystem has been described in a handful of species spanning only three Formicidae subfamilies (*Camponotus* spp*.* [24,55], *A. vollenweideri* [20], *O. biroi* [11], *E. burchellii* [12]), the present study provides an evolutionary perspective across the ant phylogeny, showing the presence of this subsystem in all eight major Formicidae clades. Beyond ants, a similar pattern of CHC-sensitive basiconic sensilla exclusively projecting into the equivalent T_B_ cluster has been described in hornets [47]. In honeybees, studies suggest that the putative equivalent of the T_B_ cluster (the T3 cluster) is predominantly innervated by basiconic sensilla OSNs but also receives inputs from sensory neurons housed in other sensilla types [46]. Whether this pattern of multi-sensilla input to T_B_ glomeruli applies to ants and wasps remains unknown, and further investigation is needed to elucidate its functional role in CHC perception.

Overall, we found the presence of basiconic sensilla on the antennae of all species, with higher density of basiconic sensilla on the distal segments, despite varying quantities across species. This observation is consistent with previous findings [11,54,57,58], supporting the role of basiconic sensilla as close-range chemoreceptors, as the distal segments of the antennae come into close proximity to other individuals during antennation [59].

Remarkably, all species also exhibited a distinct separation between the main-AL and the T_B_ cluster, forming two glomerular rings on optical sections, with the T_B_ cluster characterized by a congregation of small, uniformly sized glomeruli. The relative size of glomeruli typically reflects the quantity of incoming OSNs, which in turn correlates with detection sensitivity to associated odourants [60–62]. As such, the smaller size of T_B_ glomeruli, indicating fewer OSNs inputs, likely reflects reduced sensitivity compared with the glomeruli of the main-AL. Consequently, T_B_ glomeruli might be well adapted for close-range detection but less effective in detecting more dispersed environmental odours. Selection pressures on the specific ability to perceive CHC profiles may therefore shape the number of T_B_ glomeruli rather than their size, emphasizing discrimination over sensitivity.

### (b) Evolutionary dynamics of ant olfactory systems

The number of OR genes and the corresponding glomerular count in the AL are believed to reflect a species' olfactory discrimination power, as odours are processed through the combinatorial activity of OSN populations [48]. Ants demonstrate extensive variation in glomeruli numbers, from 198 in *Cataglyphis fortis* [63] to 630 in *Apterostigma* cf. *mayri* [10], which is at least 3−4 times higher than in most holometabolous insects whose olfactory pathway has been described, such as *Drosophila melanogaster* with 52 [64], *Aedes aegypti* with 50 [65] or *Manduca sexta* with 65 [66]. This high number is believed to heighten ants' olfactory discrimination abilities, supporting their prominent collective behaviours [40]. Nonetheless, such substantial glomerular counts might have either expanded in response to increasing social complexity or, conversely, been a pre-existing feature that facilitated the radiation of social lineages [2,67]. Our ancestral state reconstructions of glomerular number suggest that an estimated 383 glomeruli were already present in the MRCA of ants, supporting the latter hypothesis. From this ancestral state, our analyses suggest that the number of glomeruli evolved dynamically to adapt to the ecological conditions of different clades, particularly the variety of chemical cues present in their environment, rather than social factors such as polygyny, colony size or foraging strategy.

The specific family of 9-exon ORs has been highlighted as particularly expanded in ants, and showing signatures of positive selection [11,19,37], suggesting that dynamic gene family evolution has accompanied the evolution of ant sociality. Additionally, several pieces of evidence hint that the size of the 9-exon OR repertoire and the associated number of glomeruli within the T_B_ cluster may adapt to varying social traits. For example, species that independently evolved social parasitism, resulting in the loss of important social traits, showed a convergent loss of ORs, particularly within the 9-exon subfamily [68,69]. As such, it was anticipated that glomeruli number (especially in the T_B_ cluster) would also show correlated evolution with social traits.

Hints of this association within the AL have also been reported, with closely related species of *Dolichoderus* ants exhibiting a higher number of T_B_ glomeruli with larger colony size [51]. However, at a broader taxonomic scale, variation in the T_B_ cluster is not significantly associated with any of the social traits we measured, including colony size. Moreover, our ancestral trait reconstruction predicts a high number of T_B_ glomeruli in the MRCA of ants, contradicting previous suggestions of a specific expansion of the CHC-related subsystem in ants [11,37]. These results rather align with later studies showing a very high number of 9-exon ORs in non-social apoid wasps outside the Formicidae family [70], suggesting that a well developed CHC-sensitive subsystem was conserved across these lineages and existed before the diversification of ants. In solitary parasitic wasps, particularly those with host-specific behaviours, females must navigate complex chemical cues during interactions with both their hosts [71] and their kleptoparasites (e.g. cuckoo wasps [72]). Similar to ants, these wasps may rely on a sophisticated CHC-sensing system, shaped by their parasitic lifestyle and competitive interactions.

Recent studies have highlighted the crucial role of serotonin in modulating olfactory behaviour across various species. In *D. melanogaster*, serotonin signalling is essential for social attraction, influencing social behaviour through the integration of sensory cues [73]. In moths, serotonin modulates sensitivity to sex pheromones, with its levels fluctuating according to the day–night cycle [74,75]. In *A. mellifera*, serotonin inhibits appetitive olfactory learning, affecting the processing of olfactory memories [76–78]. In ants, we suggest that serotonergic innervation of the entire AL is ancestral to Formicidae, with distinct serotonergic neurons innervating the main-AL and T_B_ cluster. Notably, the branch innervation of the T_B_ cluster has been repeatedly lost in different ant subfamilies. This functional specialization may reflect the differentiation of the subregions, but the exact significance of this loss for olfactory modulation remains to be elucidated.

### (c) Ecological correlates of olfactory adaptation in ants

When assessing the scaling relationship of AL subsystems as an indicator of relative investment in perceiving distinct sets of olfactory stimuli, we observed a general covariation suggesting that ants generally maintain a consistent scaling relationship between the T_B_ cluster and the main-AL. However, *P. boopis* and Dorylinae species exhibit a notable deviation from this trend, characterized by a disproportionately high number of T_B_ glomeruli. This drastic expansion has been linked with predatory myrmecophagy, nomadism and cyclical reproduction within the Dorylinae [12]. By contrast, *Pseudomyrmex* are herbivorous and omnivorous ants, residing in moderately sized colonies [79], predominantly in arboreal environments, which does not align with these interpretations. In addition to prey-related chemical cues, long-chain hydrocarbons from food sources such as pollen, and seed elaiosomes may also be detected by the T_B_ subsystem. Consequently, foraging strategies that involve detecting these lipophilic odours could also have influenced the evolution of the T_B_ cluster in non-predatory species.

Testing ecological factors in phylogeny-controlled generalized linear mixed models (GLMMs), we found evidence of a potential role of CHC complexity in influencing the relationship between the T_B_ cluster and the main-AL. Species with a lower diversity of CHC classes in their chemical profiles exhibit a higher number of glomeruli in the T_B_ cluster. This correlation implies that the sophistication of the olfactory system may compensate for a reduced discriminative value in chemical signatures, thereby enabling accurate recognition among a smaller set of informative compounds within the CHC profile. Our behavioural experiments further support this hypothesis by demonstrating that despite variation in the number of glomeruli in the T_B_ cluster, different species perform similarly in simple nestmate discrimination tasks. Therefore, the complexity of CHC profiles likely interacts with the neuronal investment in the T_B_ cluster to maintain consistent behavioural responses in nestmate recognition, despite interspecific variation in colony-specific CHC profiles. However, it is important to note that CHC complexity exhibited a relatively low explanatory power in our analyses. Hence, in addition to other ecological factors, future research should explore how the complexity of sensory systems and the chemical environment (CHC profile, but also nest and food odours) interact to drive adaptations within communication systems and influence social evolution.

Thus, ants’ remarkable evolutionary radiation, which led to a wide array of social structures and life history traits, was supported by an already complex olfactory system. In most cases, this evolution maintained a strict balance between the main-AL, thought to be responsible for detecting general odourants, and the T_B_ cluster, assumed to be specialized in recognizing social cues. However, recent deorphanization of certain 9-exon ORs challenges this strict functional separation, as these receptors respond not only to CHCs but also to more general odourants [26,27]. Despite these insights, how these two AL regions process olfactory information at the neuronal level remains largely unexplored. Future central electrophysiological and imaging studies will be essential to further investigate potential functional specialization in odour processing between the two clusters and to clarify the significance of the T_B_ cluster adaptations.

## 4. Material and methods

Detailed methods for sample preparation, imaging, behavioural assays, chemical analysis and statistical tests are provided in electronic supplementary material, Methods. In brief, 14 ant species from eight subfamilies were studied, including nine species from lab rearing and five collected in the wild. In all cases, ant workers were anaesthetized on ice before heads and antennae were separated, and bodies were stored in 95% ethanol. To investigate phenotypic variation of the olfactory system, antennae were preserved in 2.5% glutaraldehyde at 4°C, and brains were dissected and preserved in methanol, following procedures in electronic supplementary material, Methods. In all but one species, antennal sensilla were analysed via scanning electron microscopy, with standard sample preparation and scanning procedures. Antennal lobe neuroanatomy was investigated using immunostaining and confocal laser scanning microscopy. Behavioural assays for nestmate recognition in *L. niger*, *M. barbarus* and *F. fusca* were recorded and scored for aggression. Chemical analyses of CHC profiles were performed via gas chromatography coupled with mass spectrometry (GC-MS). Phylogenetic analyses used branch lengths inferred from phylogenomic studies [80–82]. Data analysis was performed using R and established statistical packages, with phylogenetic comparisons and evolutionary analyses exploring relationships between antennal morphology, neuroanatomy, chemical complexity and behaviour. All specific details of materials used, such as reagents, animal models or software are detailed in electronic supplementary material, Methods.

## Data Availability

All datasets supporting this study are publicly available in the online electronic supplementary material [83] and the Dryad Digital Repository [43].
